# Association Between Sugar-Sweetened Beverage Consumption and Executive Function Among Chinese Tibetan Adolescents at High Altitude

**DOI:** 10.3389/fnut.2022.939256

**Published:** 2022-06-27

**Authors:** Feng Zhang, Xiaojian Yin, Yuan Liu, Ming Li, Xiaoying Gui, Cunjian Bi

**Affiliations:** ^1^Key Laboratory of Adolescent Health Assessment and Exercise Intervention of Ministry of Education, East China Normal University, Shanghai, China; ^2^College of Physical Education and Health, East China Normal University, Shanghai, China; ^3^College of Economics and Management, Shanghai Institute of Technology, Shanghai, China; ^4^Lhasa Beijing Experimental Middle School, Lhasa, China

**Keywords:** Chinese Tibetan adolescents, sugar-sweetened beverages, inhibition, working memory, cognitive flexibility

## Abstract

**Objective:**

To estimate the association between sugar-sweetened beverage (SSB) consumption and executive function (EF) among Chinese Tibetan adolescents.

**Method:**

Using three stages by stratified cluster sampling, 1,427 Chinese Tibetan adolescents were recruited from Tibet, China. SSB consumption status was obtained by questionnaires and the three core EFs (inhibition, working memory, and cognitive flexibility) were tested by a modified Eriksen flanker task, N-back shift, and a more-odd shifting task. One-way ANOVA or Chi-square test was used to compare SSB consumption in different categories. Taking the SSB consumption 0 time/week group as the reference, general linear regression (for continuous variable) or Logical regression (classified variable) in three Models was conducted to analyze the relationship between SSB consumption and EF for Chinese Tibetan children and adolescents.

**Result:**

After adjustment of all the covariant in Model 2, all the EF indexes were higher in Chinese Tibetan adolescents with SSB consumption ≥2 times/week than that with SSB consumption of 0 times/week by 21.33 ms (95%*CI*: 6.72, 35.93), 8.21 ms (95%*CI*: 7.06, 9.35), 90.46 ms (95%*CI*: 28.69, 152.23), 147.61 ms (95%*CI*: 81.42, 213.80), 116.18 ms (95%*CI*: 74.48, 157.87), 112.41 ms (95%*CI*: 71.30, 153.52) for incongruent RT, RT difference in incongruent and congruent, 1-back RT, 2-back RT, Heterogeneous RT, RT difference in Heterogeneous and Homogeneous respectively.

**Conclusions:**

The results suggested that SSB consumption was associated with poorer EF in Chinese Tibetan adolescents. SSB consumption should be controlled for healthy brain development of Chinese Tibetan adolescents.

## Introduction

Located on the Qinghai-Tibet Plateau, Xizang (Tibet) is a remote and low-income area in China, with GDP ranking last in 2021 (1). According to the sixth National census, there are 6.5 million permanent Tibetan residents on the Qinghai-Tibet Plateau, of whom 15% live between 2,500 and 3,000 meters above sea level, 75% live between 3,000 and 4,000 m above sea level, and 10% live above 4,000 m (2). With an average high of 4,000 m above sea level, Chinese Tibetan adolescents are suffering from hypoxia, which affected their body and brain development, including executive function (EF) (3).

Researches on EF has flourished since 2000 in developmental psychology (4). Considered as “air traffic control system of the brain,” EF is a collection of top-down control processes used when going on automatic or relying on instinct or intuition would be ill-advised, insufficient, or impossible, such as attentional control, working memory, inhibition, and problem-solving (5). Several studies have found concurrent or longitudinal relations between children's EF and diverse skills, including academic achievement (6), social (7), logical (8), and biological reasoning (9, 10)_._ Several studies have suggested the poor EF of adolescents at high altitudes because of the sensitivity of the brain to environmental hypoxia (11, 12). To our best knowledge, we did not find the data on EF for Chinese Tibetan adolescents in Tibet, China.

As one of the factors influencing EF and underlying brain developmental processes in children, a balanced diet may provide an effective way to promote EF (13). Sugar-sweetened beverages (SSB), a class of very popular non-alcoholic beverages throughout the world, are characterized by high added sugar content, especially fructose-containing sugar (14). SSB was considered to be an important risk factor for obesity, type 2 diabetes mellitus, cardiovascular disease, mortality, and certain cancers worldwide (15, 16). In childhood, evidence supports links between SSB consumption and unhealthy weight gain, as well as other untoward health outcomes, such as dental caries, the earlier timing of puberty, higher blood pressure, and hyperactivity/inattention symptoms (17).

In recent decades, the global production and consumption of SSB have been increasing (18). SSB consumption has declined or plateaued in most western high-income countries since the early 2000s, while in many low-income and middle-income countries, the intake of SSB consumption is increasing, as widespread urbanization and economic development have increased the availability of these beverages (19, 20). In China, it was reported that the production of SSB exceeded 180 million tons in 2017, which was 440 times that of 1992 (21). Compared with 2014, the proportion of SSB non-drinkers among Chinese Tibetan primary school students in Lhasa decreased from 15.16 to 2.47 % in 2019 (22), which was lower than their peers in South China (34.7%) (23). Meanwhile, racial/ethnic disparities in SSB consumption were observed in multi-ethnic areas (24, 25). Verzeletti also emphasized that the ethnic background differences may have an impact on parental beliefs regarding the child's SSB consumption or on rules restricting the intake of SSB by the child (26).

Evidence from a systematic review suggested that less-healthy foods such as SSB were inversely associated with EF (27). The limited studies focusing on this topic has confirmed the association between SSB consumption and poorer EF among children in South China (28). Besides, population-based studies also observed associations between SSB and declined intelligence (29), and poor poorer cognitive performance (30, 31). Nevertheless, the association between SSB consumption and EF among Chinese Tibetan adolescents has been rarely investigated. Given the large population of Chinese Tibetan, the present study aimed to estimate the association between SSB consumption and EF among Chinese Tibetan adolescents.

## Methods

### Data Sources and Participants Recruitment

Data were obtained from a cross-sectional study of Chinese Tibetan adolescents in Tibet, China and the research was conducted from August 2019 to December 2020. The participants in this study should be: (1) Chinese Tibetan middle school students with IQ > 90 according to the Wechsler intelligence scale; (2) without physical disability. (3) No color blindness or color weakness; (4) right-handed (32, 33); (5) without depression, anxiety, and other adverse psychological emotions. (6) Born and grew up in Tibet, China with their parents as indigenous Chinese Tibetan.

**Figure 1:**
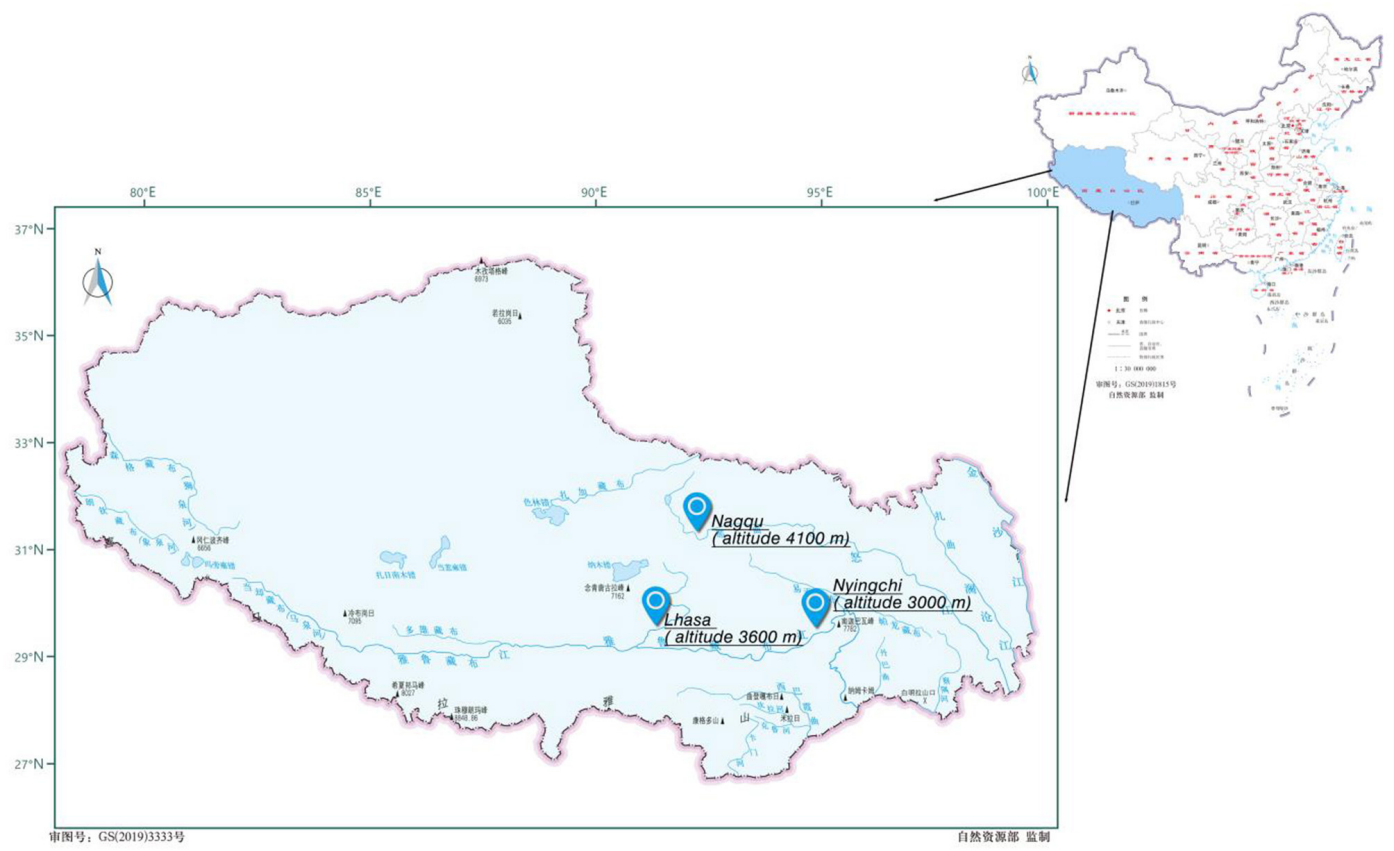
Map of the three cities in Tibet of China.

**Figure 2:**
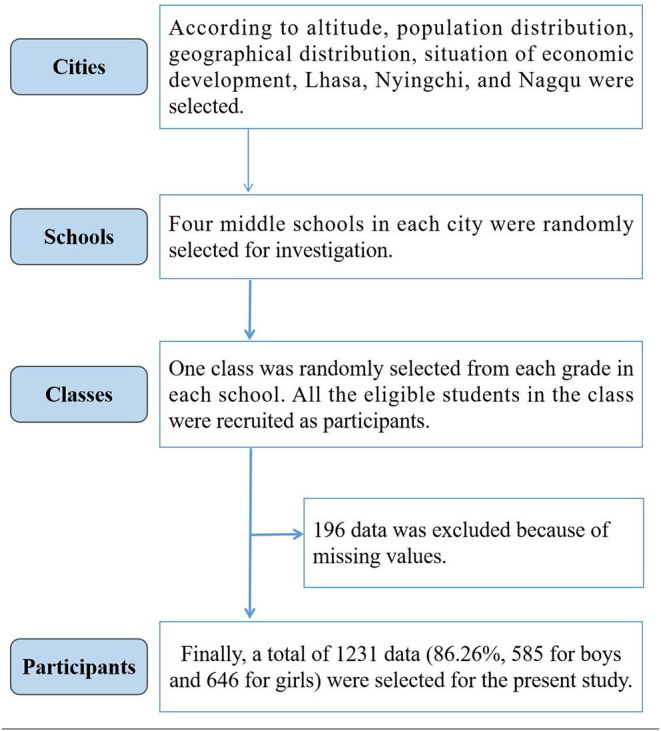
Flowchart of participant inclusion for Chinese Tibetan adolescents.

The recruitment procedure includes three stages by stratified cluster sampling. Firstly, according to altitude, population distribution, geographical distribution, and the situation of economic development of Tibet in China, three cities (Lhasa, Nyingchi, and Nagqu) were selected (Figure 1). Secondly, four middle schools in each city were randomly selected for investigation. Thirdly, taking class as the smallest unit of cluster sampling, one class was randomly selected from each grade in each school. All the eligible students in the class were recruited as participants. A total of 1,427 Chinese Tibetan adolescents were recruited and 196 data were excluded because of missing values. Finally, 1,231 data were effective for the present study (86.26%; Figure 2).

This study was approved by the Human Experimental Ethics Committee of the East China Normal University (Approval No.: HR0782020). Written informed consent was obtained from the students and their parents before the study. The names of participants were coded to protect their privacy.

### Procedure

Before the test, all the team members, composed of graduate students majoring in human sports science, were required to be trained until they were qualified for the test. A self-administered questionnaire collecting demographic information, data on SSB, and dietary intake was completed by the adolescents independently over a 40-min period in classrooms after school. To make sure each item of the questionnaire fully understood, the questionnaire was translated into Tibetan. The team members explained every item in detail to students when they administered the questionnaires and were available all the time for any questions raised by students. When completed, the questionnaires were withdrawn on the spot.

#### Sugar-Sweetened Beverage

Data on SSB consumption were obtained by questionnaires extracted from the Chinese National Survey on Students' Constitution and Health (CNSSCH) (34) and its validation and reliability have been confirmed in previous studies (35, 36). The SSB consumption was assessed by asking them the frequency of SSB consumption from the question “In the past 7 days, how many times did you drink SSB such as Coke, Sprite, Natural fruit juices, Nutrition Express, Red Bull?” (0, 1, 2, 3, 4, ≥5). The frequency of SSB consumption was aggregated and categorized into three groups (0 time/week, 1 time/week, and ≥2 times/week) to ensure an adequate number of participants in each group.

#### Executive Function

In the present study, three core EFs (inhibition, working memory, and cognitive flexibility) were tested on the computer by a test tool developed by Chen et al. (37), which has been used in many previous studies (38–41). A modified Eriksen flanker task was used to assess the inhibition aspect of EF (42), and the response times (RT, ms) in the congruent and incongruent trials were recorded as result. The RT difference between incongruent and congruent trials was used as an index of inhibition. Shorter RT and RT differences between incongruent and congruent indicated better performance. The working memory aspect of EF was assessed by 1-back and 2-back tasks. The RT on correct trials was recorded as result and shorter RT reflected better performance. Adapted from Hillman et al. (43) and Salthouse et al. (44), a more-odd shifting task was employed to assess the cognitive flexibility aspect of EF. The RT difference between the heterogeneous and homogeneous blocks was used to estimate cognitive flexibility. Each test was conducted according to the details introduced in the previous paper (45). The stimulus presentation and response data collection were conducted using E-Prime software 1.1 (Psychology Software Tools Inc., Pittsburgh, USA).

The test was conducted in a computer classroom with at least one Chinese Tibetan teacher present for better communication. Before the test, the Chinese Tibetan teachers, who in charge of the test, were trained uniformly until they were qualified. A video explaining how to do the test was also played before the test to make the students better understand until the students were familiar with the test.

#### Covariant

Results from many studies have demonstrated that EF can be affected by obesity rate (46–48), dietary intake (49), physical activity (50), and VO_2max_ (51, 52). Therefore, the present study took sociodemographic and dietary intake information, BMI, WC, Moderate-to-Vigorous Physical Activity (MVPA), and VO_2max_ as covariant.

Sociodemographic information including age (years), sex (boys or girls), siblings (0 or ≥1), father's and mother's education (without education,elementary school,junior middle school, senior middle school, college, or above) was obtained by questionnaires. Information on dietary intake was also collected by questionnaires separately with three questions “In the past 7 days, how many times did you have breakfast? (0, 1, 2, 3, 4, 5, 6, 7)”; “In the past 7 days, how many times did you have eggs or egg products? (0, 1, 2, 3, 4, 5, 6, 7)”; “In the past 7 days, how many times did you have milk or dairy products? (0, 1, 2, 3, 4, 5, 6, 7)”. Physical activity status was obtained by the following two questions: “In the past 7 days, how many times did you have physical activity (Moderate-intensity physical activity and Vigorous-intensity physical activity) on school days and weekends, respectively?” The students were asked to respond in the blanks. Moderate-intensity physical activity refers to activity that requires moderate effort, and makes you feel a little breathless, sweaty, or tired, such as cycling at normal speed, brisk walking, skating, etc. Vigorous-intensity physical activity refers to activity that requires a lot of effort, feeling breathless, sweaty, or very tiring, such as heavy lifting, running fast, playing with balls hardly, or cycling fast. If they answered more than 0 times, they were further asked about the duration each time, “On average, how long does each activity last?” Height, weight, and waist circumference (WC) were measured with participants lightly dressed and barely feet. Body mass index (BMI) was calculated as weight (in kilograms) over height (meters) square. The VO_2max_ was estimated by 20 m SRT and the details measurement of 20 m SRT was provided in our previous study (53). All the instruments were calibrated to ensure the accuracy of the test. The test was carried out at the same time every day to reduce the deviation.

### Statistical Analyses

For the continuous variables, mean and standard deviation (M ± SD) were used to express the result, and One-way ANOVA was used to compare SSB consumption in different categories. For the classified variable, data were expressed by percentage, and the Chi-square test was used to compare SSB consumption in different categories.

Taking the SSB consumption 0 time/week group as the reference, general linear regression (for continuous variable) or Logical regression (classified variable) was conducted to analyze the relationship between SSB consumption and EF for Chinese Tibetan adolescents. Three models (Crude Model, Model 1, Model 2)were used: Crude Model was conducted without adjustment; Model 1 was conducted after adjusting age, sex, siblings, parental education, BMI, WC, MVPA, and VO_2max_; Based on Model 1, Model 2 included breakfast, egg or egg products, and milk or dairy-products as additional control variables. We set dummy variables for SSB consumption and took them as continuous variables to estimate the dose-response relationship between SSB consumption and EF. The mean and standard deviation (SD) of RT for the three core EFs were calculated by age and sex. Executive dysfunction was defined as ≥1 SD from the mean.

All analyses were performed using IBM SPSS (version 25.0; IBM Inc., Armonk, NY) and GraphPad Prism 8.0.2 (GraphPad Software, Inc., CA). The level of statistical significance was set at a two-tailed P <0.05.

## Result

Among 1,231 Chinese Tibetan adolescents aged 13–18 from Tibet of China, 585 (47.5%) were boys with an average age of (15.77 ± 1.68) years. There are 634 (51.50%) adolescents with SSB consumption 1 time/week, and 409 (33.23%) adolescents with SSB consumption ≥2 time/week. Compared with non-SSB consumption, adolescents with SSB consumption ≥1 time/week have lower parental education, fewer breakfast times per week, higher BMI and waist circumference, and lower MVPA and VO_2max_ levels (*P* < 0.05; Table 1).

**Table 1:** The SSB consumption status of Chinese Tibetan adolescents in Tibet, China.

**Characteristics**	**Total sample**	**SSB consumption**			**χ^2^/*F***	***P*-Value**
		**0 time/week**	**1 time/week**	**≥2 time/week**		
*N*	1,231	188 (15.27)	634 (51.50)	409 (33.23)	363.58	<0.001
Age	15.77 ± 1.68	15.95 ± 1.81	15.93 ± 1.65	15.44 ± 1.61	12.13	<0.001
**Sex**						
Boys	585 (47.5)	102 (54.3)	262 (41.3)	221 (54.0)	20.14	<0.001
Girls	646 (52.5)	86 (45.7)	372 (58.7)	188 (46.0)		
**Siblings**						
0	198 (16.1)	29 (15.4)	97 (15.3)	72 (17.6)	1.05	0.59
≥1	1,033 (83.9)	159 (84.6)	537 (84.7)	337 (82.4)		
**Father's education**						
Elementary school and below	683 (63.2)	95 (58.3)	366 (64.7)	222 (63.2)	13.16	0.01
Middle school	268 (24.8)	36 (22.1)	135 (23.9)	97 (27.6)		
College and above	129 (11.9)	32 (19.6)	65 (11.5)	32 (9.1)		
**Mother's education**						
Elementary school and below	775 (72.2)	93 (57.8)	432 (75.9)	250 (72.9)	38.95	<0.001
Middle school	164 (15.3)	25 (15.5)	78 (13.7)	61 (17.8)		
College and above	134 (12.5)	43 (26.7)	59 (10.4)	32 (9.3)		
**Breakfast**						
≤ 1 time/week	35 (2.8)	9 (4.8)	12 (1.9)	14 (3.4)	13.85	0.01
2–4 times/week	67 (5.4)	9 (4.8)	25 (3.9)	33 (8.1)		
≥5 times/week	1,129 (91.7)	170 (90.4)	597 (94.2)	362 (88.5)		
**Eggs or egg products**						
≤ 1 time/week	356 (28.9)	54 (28.7)	192 (30.3)	110 (26.9)	4.58	0.33
2–4 times/week	562 (45.7)	79 (42.0)	281 (44.3)	202 (49.4)		
≥5 times/week	313 (25.4)	55 (29.3)	161 (25.4)	97 (23.7)		
**Milk or dairy-products**						
≤ 1 time/week	349 (28.4)	68 (36.2)	179 (28.2)	102 (24.9)	9.21	0.06
2–4 times/week	619 (50.3)	79 (42.0)	321 (50.6)	219 (53.5)		
≥5 times/week	263 (21.4)	41 (21.8)	134 (21.1)	88 (21.5)		
BMI	20.49 ± 2.37	19.74 ± 1.79	20.4 ± 2.46	20.98 ± 2.37	19.31	<0.001
WC	68.56 ± 7.22	67.38 ± 8.00	67.97 ± 6.90	70.01 ± 7.12	13.06	<0.001
MVPA	41.81 ± 23.48	60.21 ± 18.45	40.25 ± 23.26	35.76 ± 21.69	82.26	<0.001
VO_2max_	37.19 ± 5.58	38.96 ± 5.48	36.85 ± 5.52	36.90 ± 5.58	11.35	<0.001

*Descriptive statistics are presented as (mean ± standard deviation) and number (percentage) for continuous and categorical.*

*SSB, sugar-sweetened beverages; BMI, body mass index; MVPA, moderate-to-vigorous physical activity; VO_2max_, maximal oxygen uptake; WC, waist circumference*.

All the EF index (including congruent RT, incongruent RT, RT difference in incongruent and congruent, 1-back RT, 2-back RT, Heterogeneous RT, Homogeneous RT, RT difference in Heterogeneous and Homogeneous) of Chinese Tibetan adolescents with different SSB consumption are significantly different (*F* = 20.44, 29.32, 174.78, 20.72, 43.87, 67.25, 9.77, and 60.23, respectively, *P* < 0.001; Table 2). Compared with non-SSB consumption, adolescents with SSB consumption ≥1 time/week have a longer reaction time, that is, the worse performance of EF (Table 2).

**Table 2:** The status of executive function for Chinese Tibetan adolescents with different SSB consumption in Tibet of China.

**RT (ms)**	**SSB**	***N***	**Mean**	**SD**	***F*-Value**	***P*-Value**
	**Consumption**					
**Inhibition**						
Congruent	0 time/week	188	745.65	94.22	20.44	<0.001
	1 time/week	634	772.52	76.96		
	≥2 times/week	409	791.86	86.64		
Incongruent	0 time/week	188	760.80	93.67	29.32	<0.001
	1 time/week	634	792.35	76.12		
	≥2 times/week	409	815.67	86.43		
Difference incongruent and congruent	0 time/week	188	15.15	3.28	174.78	<0.001
	1 time/week	634	19.84	6.31		
	≥2 times/week	409	23.81	4.53		
**Working memory**						
1-back	0 time/week	188	927.03	351.95	20.72	<0.001
	1 time/week	634	938.24	311.75		
	≥2 times/week	409	1,056.71	290.49		
2-back	0 time/week	188	960.10	360.41	43.87	<0.001
	1 time/week	634	1,075.45	380.06		
	≥2 times/week	409	1,226.01	275.48		
**Cognitive flexibility**						
Heterogeneous	0 time/week	188	1,017.51	209.75	67.25	<0.001
	1 time/week	634	1,100.70	253.78		
	≥2 times/week	409	1,236.58	221.42		
Homogeneous	0 time/week	188	737.48	133.50	9.77	<0.001
	1 time/week	634	741.79	114.80		
	≥2 times/week	409	771.56	107.75		
Difference in heterogeneous and homogeneous	0 time/week	188	280.04	179.29	60.23	<0.001
	1 time/week	634	358.90	228.52		
	≥2 times/week	409	465.02	178.77		

*Descriptive statistics are presented as (mean ± SD).*

*SSB, sugar-sweetened beverages; N, number of the sample; SD, standard deviation*.

There is a significant correlation between SSB consumption and EF for Chinese Tibetan adolescents in Tibet of China (Table 3). After adjustment in Model 2, all the EF index were higher in Chinese Tibetan adolescents with SSB consumption ≥2 time/week than that with SSB consumption 0 time/week by 21.33 ms (95%*CI*: 6.72, 35.93), 8.21 ms(95%*CI*: 7.06, 9.35), 90.46 ms (95%*CI*: 28.69, 152.23), 147.61 ms (95%*CI*: 81.42, 213.80), 116.18 ms (95%*CI*: 74.48, 157.87), 112.41 ms (95%*CI*: 71.30, 153.52) for incongruent RT, RT difference in incongruent and congruent, 1-back RT, 2-back RT, Heterogeneous RT, RT difference in Heterogeneous and Homogeneous, respectively (*P* <0.05).

**Table 3:** The multiple linear regression of executive function for Chinese Tibetan adolescents with different SSB consumption (*n* = 1,231).

**RT (ms)**	**Estimates (95% Confidence Interval)**		
	**Crude Model**	**Model 1**	**Model 2**
**Inhibition**			
**Congruent**			
0 time/week	0 (Reference)	0 (Reference)	0 (Reference)
1 time/week	26.87 (13.33, 40.40)^a^	7.56 (−5.67, 20.79)	6.59 (−6.64, 19.82)
≥2 times/week	46.21 (31.85, 60.57)^a^	14.16 (−0.58, 28.89)	13.12 (−1.59, 27.83)
*P* for trend	<0.001	<0.001	<0.001
**Incongruent**			
0 time/week	0 (Reference)	0 (Reference)	0 (Reference)
1 time/week	31.55 (18.11, 44.99)^a^	12.15 (−0.98, 25.28)	11.21 (−1.92, 24.34)
≥2 times/week	54.87 (40.61, 69.13)^a^	22.32 (7.69, 36.95)^a^	21.33 (6.72, 35.93)^a^
*P* for trend	<0.001	<0.001	<0.001
**RT difference in incongruent and congruent**			
0 time/week	0 (Reference)	0 (Reference)	0 (Reference)
1 time/week	4.69 (3.81, 5.56)^a^	4.60 (3.57, 5.62)^a^	4.62 (3.59, 5.65)^a^
≥2 times/week	8.66 (7.73, 9.59)^a^	8.16 (7.02, 9.30)^a^	8.21 (7.06, 9.35)^a^
*P* for trend	<0.001	<0.001	<0.001
**Working memory**			
**1-back**			
0 time/week	0 (Reference)	0 (Reference)	0 (Reference)
1 time/week	11.20 (−39.54, 61.95)	−14.32 (−69.75, 41.12)	−14.28 (−69.81, 41.25)
≥2 times/week	129.67 (75.83, 183.51)^a^	89.91 (28.15, 151.67)^a^	90.46 (28.69, 152.23)^a^
*P* for trend	<0.001	<0.001	<0.001
**2-back**			
0 time/week	0 (Reference)	0 (Reference)	0 (Reference)
1 time/week	115.35 (59.04, 171.66)^a^	53.24 (−5.95, 112.43)	53.48 (−6.02, 112.99)
≥2 times/week	265.92 (206.17, 325.66)^a^	147.40 (81.46, 213.34)^a^	147.61 (81.42, 213.80)^a^
*P* for trend	<0.001	<0.001	<0.001
**Cognitive flexibility**			
**Heterogeneous**			
0 time/week	0 (Reference)	0 (Reference)	0 (Reference)
1 time/week	83.18 (44.56, 121.80)^a^	14.69 (−22.70, 52.07)	14.54 (−22.94, 52.03)
≥2 times/week	219.06 (178.09, 260.04)^a^	116.93 (75.28, 158.57)^a^	116.18 (74.48, 157.87)^a^
*P* for trend	<0.001	<0.001	<0.001
**Homogeneous**			
0 time/week	0 (Reference)	0 (Reference)	0 (Reference)
1 time/week	4.32 (−14.52, 23.15)	−11.77 (−30.82, 7.29)	−11.71 (−30.76, 7.35)
≥2 times/week	34.09 (14.10, 54.07)^a^	3.57 (−17.66, 24.79)	3.76 (−17.43, 24.95)
*P* for trend	<0.001	<0.001	<0.001
**RT difference in Heterogeneous and Homogeneous**			
0 time/week	0 (Reference)	0 (Reference)	0 (Reference)
1 time/week	78.87 (45.30, 112.43)	26.46 (−10.33, 63.25)	26.25 (−10.71, 63.21)
≥2 times/week	184.98 (149.37, 220.59)	113.36 (72.37, 154.34)^a^	112.41 (71.30, 153.52)^a^
*P* for trend	<0.001	<0.001	<0.001

*Crude Model: without adjustment; Model 1: adjusting age, sex, siblings, parental education, BMI, WC, MVPA, and VO_2max_; Model 2: based on Model 1, including breakfast, egg, or egg products, and milk or dairy products as additional control variables.*

*^a^Indicate P <0.001*.

Table 4 shows the logistic regression of executive dyfunction for Chinese Tibetan adolescents with different SSB consumption. After adding additional control variables in Model 2, Chinese Tibetan adolescents with SSB consumption ≥2 times/week perform poorer on the three core EFs (inhibition, working memory, cognitive flexibility) than that with SSB consumption 0 time/week [*OR* = 5.91, (95%*CI*: 2.78, 12.59)], [2.98, (95%*CI*: 1.40, 6.34)], [2.80, (95% *CI*:1.16, 6.74)], respectively (*P* <0.05).

**Table 4:** The logistic regression of executive function for Chinese Tibetan adolescents with different SSB consumption (*n* = 1,231).

**Executive dysfunction**	**Odds ratio (95% Confidence Interval)**		
	**Crude Model**	**Model 1**	**Model 2**
**Inhibition dysfunction**			
0 time/week	1.00 (Reference)	1.00 (Reference)	1.00 (Reference)
1 time/week	4.20 (2.16, 8.18)^a^	3.12 (1.51, 6.48)^a^	3.10 (1.49, 6.44)^a^
≥2 times/week	7.22 (3.69, 14.13)^a^	5.93 (2.79, 12.61)^a^	5.91 (2.78, 12.59)^a^
*P* for trend	<0.001	<0.001	<0.001
**Working memory dysfunction**			
**1-back**			
0 time/week	1.00 (Reference)	1.00 (Reference)	1.00 (Reference)
1 time/week	0.82 (0.54, 1.24)	0.68 (0.41, 1.13)	0.71 (0.42, 1.19)
≥2 times/week	1.45 (0.95, 2.21)	1.29 (0.75, 2.22)	1.37 (0.79, 2.36)
*P* for trend	<0.001	<0.001	<0.001
**2-back**			
0 time/week	1.00 (Reference)	1.00 (Reference)	1.00 (Reference)
1 time/week	2.91 (1.68, 5.02)^a^	2.67 (1.30, 5.47)^a^	2.75 (1.34, 5.65)^b^
≥2 times/week	3.30 (1.88, 5.78)^a^	2.89 (1.36, 6.14)^a^	2.98 (1.40, 6.34)^a^
*P* for trend	<0.001	<0.001	<0.001
**Cognitive flexibility dysfunction**			
0 time/week	1.00 (Reference)	1.00 (Reference)	1.00 (Reference)
1 time/week	4.31 (2.06, 9.03)^a^	2.22 (0.96, 5.16)	2.32 (0.99, 5.46)
≥2 times/week	6.90 (3.28, 14.53)^a^	2.67 (1.12, 6.36)^b^	2.80 (1.16, 6.74)^b^
*P* for trend	<0.001	<0.001	<0.001

*Crude Model: without adjustment; Model 1: adjusting age, sex, siblings, parental education, BMI, WC, MVPA, and VO_2max_; Model 2: based on Model 1, including breakfast, egg, or egg products, and milk or dairy products as additional control variables.*

*^a^Indicate P <0.01.*

*^b^Indicate P <0.05*.

## Discussion

The present study cross-sectionally analyzed the association between SSB consumption and EF in Chinese Tibetan adolescents in high-altitude areas of China. The result showed that the SSB consumption of Chinese Tibetan adolescents in high-altitude areas was related to the poor performance of the EF. After adjusting for demographic factors, sociodemographic information, dietary behaviors, and some physical status, there was still a significant correlation between SSB consumption and EF in Chinese Tibetan adolescents. At the same time, our study also observed that SSB consumption was associated with a higher risk of executive dysfunction.

Our study showed that 84.73% of Chinese Tibetan adolescents consumed at least one time of SSB in the past week, which was higher than American adolescents (60.7%) (54). Over the past decades, excessive SSB consumption is spreading into low and middle-income countries, leading to an increase in chronic non-communicable diseases, cancer, and all-cause mortality, bringing a huge medical burden to the country (55, 56). It was reported that SSB consumption of children and adolescents was associated with parental SSB consumption patterns and accessibility of SSB consumption (57), frequency of fast-food consumption (58), and time spent watching television or viewing advertisements (59). Rodent studies suggest that SSB consumption may activate a glucocorticoid-metabolic-brain-negative feedback pathway, which may turn off the stress response and thereby reinforce habitual SSB overconsumption (60). Hence, targeted efforts such as taxing SSB and increasing knowledge of SSB are needed to reduce intake of SSB consumption are needed.

Similar to our findings, Gui et al. also found out that SSB consumption was associated with poorer EF among children in Guangzhou, China, and the global executive index of children with SSB consumption ≥2 times/week increased by 1.62 times compared with non-SSB consumption children (28). A study of children and adolescents in the United States showed that an increase in daily SSB consumption was associated with a 2.4-point decline in the intelligence of children and adolescents as assessed by the Kaufman Brief Intelligence Test (29). Though the association was observed between SSB consumption and EF, the bi-directional associations between them remain unclear. Obesity researchers emphasize the significance of executive-control systems for explaining the occurrence of non-homeostatic forms of dietary behavior and modulating cravings for and consumption of high-calorie foods (61). While research from other disciplines suggested SSB was inversely associated with EF (27). Hence, more longitudinal studies are needed to explore the causal relationship between them.

A systematic review suggested that the relation between EF and dietary intake is equivocal (49). In addition, a study focused on school-aged children aged 8–10 years in the United States found no association between SSB consumption and working memory, academic performance, cognition, and inhibitory control (62). The wide variety of measures used to assess EF and dietary intake may play in the relation between EF and dietary intake, making the overall interpretation of the literature more complicated. For example, EF can be used by questionnaires such as Behavior Rating Inventory of Executive Function or computerized tests (Computerized Dots Task, Computerized Neuro-psychological Test). Dietary intake can be measured by Food Frequency Questionnaire or Lab-Based Food Task. Besides, the non-uniform covariant may also affect the result. In sum, the relation between EF and SSB needs to be further confirmed.

This study has some strengths and limitations. The strength is that we controlled several covariant such as sociodemographic information (age, sex, siblings, father's and mother's education), information on dietary intakes (eggs, milk, breakfast), and physical status (BMI, WC, MVPA, VO_2max_,). However, the cross-sectional analysis of the present study can't decide a causal relationship. A prospective cohort study is needed in the future. Besides, we used self-report SSB consumption, which are inevitably affected by the recall ability. Meanwhile, the information on volumes of SSBs consumption by adolescents were not collected. At last, the impact of confounding variables on EF was not included in the study.

## Conclusions

In conclusion, this study analyzed the relationship between SSB consumption and EF for Chinese Tibetan adolescents at high altitudes and concluded that SSB consumption is associated with poorer EF performance and executive dysfunction. Given the large population of Chinese Tibetan and the brain damage caused by hypoxia at high altitudes, it is necessary to make targeted efforts to reduce SSB consumption of Chinese Tibetan adolescents in high altitude, such as taxing SSB, increasing knowledge of SSB, or environmental interventions that alter the physical or social environment in which individuals make beverage choices. Longitudinal studies and clinical trials are further needed to clarify the direction of causality and to investigate the underlying mechanism.

## Data Availability Statement

The raw data supporting the conclusions of this article will be made available by the authors, without unduereservation.

## Ethics Statement

The study was conducted according to the guidelines of the Declaration of Helsinki and approved by the Human Experimental Ethics Committee of the East China Normal University (Approval No.: HR0782020). Written informed consent to participate in this study was provided by the participants' legal guardian/next of kin.

## Author Contributions

Conceptualization: FZ, XY, and CB. Methodology: YL and ML. Validation, supervision, and funding acquisition: XY. Formal analysis and visualization: FZ. Investigation: FZ and ML. Resources, writing—original draft preparation, and project administration: ML. Data curation: XG. Writing—review and editing: FZ and YL. All authors have read and agreed to the published version of the manuscript.

## Funding

This work was supported by the Shanghai Planning Project of Philosophy and Social Science (Award No.: 2020BTY001).

## Conflict of Interest

The authors declare that the research was conducted in the absence of any commercial or financial relationships that could be construed as a potential conflict of interest.

## Publisher's Note

All claims expressed in this article are solely those of the authors and do not necessarily represent those of their affiliated organizations, or those of the publisher, the editors and the reviewers. Any product that may be evaluated in this article, or claim that may be made by its manufacturer, is not guaranteed or endorsed by the publisher.
